# Heat shock protein 70 and AMP‐activated protein kinase contribute to 17‐DMAG‐dependent protection against heat stroke

**DOI:** 10.1111/jcmm.12881

**Published:** 2016-05-31

**Authors:** Yung‐Chieh Tsai, Kwok‐Keung Lam, Yi‐Jen Peng, Yen‐Mei Lee, Chung‐Yu Yang, Yi‐Ju Tsai, Mao‐Hsiung Yen, Pao‐Yun Cheng

**Affiliations:** ^1^ Department of Obstetrics and GynecologyChi‐Mei Medical CenterTainanTaiwan; ^2^ Department of MedicineTaipei Medical UniversityTaipeiTaiwan; ^3^ Department of Sport ManagementChia Nan University of Pharmacy and ScienceTainanTaiwan; ^4^ Department of PharmacologyTaipei Medical UniversityTaipeiTaiwan; ^5^ Department of AnesthesiologyCatholic Mercy HospitalHsinchuTaiwan; ^6^ Department of PathologyTri‐Service General Hospital and National Defense Medical CenterTaipeiTaiwan; ^7^ Department of PharmacologyNational Defense Medical CenterTaipeiTaiwan; ^8^ Department of Physiology and BiophysicsNational Defense Medical CenterTaipeiTaiwan

**Keywords:** heat stroke, heat shock protein 70, AMP‐activated protein kinase, 17‐DMAG, autophagy, mortality

## Abstract

Heat shock protein 70 (Hsp70) preconditioning induces thermotolerance, and adenosine monophosphate (AMP)‐activated protein kinase (AMPK) plays a role in the process of autophagy. Here, we investigated whether 17‐dimethylaminoethylamino‐17‐demethoxy‐geldanamycin (17‐DMAG) protected against heat stroke (HS) in rats by up‐regulation of Hsp70 and phosphorylated AMPK (pAMPK). To produce HS, male Sprague–Dawley rats were placed in a chamber with an ambient temperature of 42°C. Physiological function (mean arterial pressure, heart rate and core temperature), hepatic and intestinal injury, inflammatory mediators and levels of Hsp70, pAMPK and light chain 3 (LC3B) in hepatic tissue were measured in HS rats or/and rats pre‐treated with 17‐DMAG. 17‐DMAG pre‐treatment significantly attenuated hypotension and organ dysfunction induced by HS in rats. The survival time during HS was also prolonged by 17‐DMAG treatment. Hsp70 expression was increased, whereas pAMPK levels in the liver were significantly decreased in HS rats. Following pre‐treatment with 17‐DMAG, Hsp70 protein levels increased further, and pAMPK levels were enhanced. Treatment with an AMPK activator significantly increased the LC3BII/LC3BI ratio as a marker of autophagy in HS rats. Treatment with quercetin significantly suppressed Hsp70 and pAMPK levels and reduced the protective effects of 17‐DMAG in HS rats. Both of Hsp70 and AMPK are involved in the 17‐DMAG‐mediated protection against HS. 17‐DMAG may be a promising candidate drug in the clinical setting.

## Introduction

Heat stroke (HS) is a life‐threatening illness characterized by an elevated core body temperature of above 40°C, resulting in multi‐organ failure such as circulatory shock and liver failure 1. Despite adequate lowering of body temperature and aggressive treatment, HS is often fatal 2. Heat shock proteins (Hsps) are a group of highly conserved proteins that function as molecular chaperones by facilitating the folding and refolding of proteins, mediating transmembrane transport of secretory proteins and targeting proteins for lysosomal degradation 3. Previous studies have established that sublethal heat stress–induced accumulation of inducible Hsp70 is necessary for acquired thermotolerance, defined as the ability of a cell or organism to become resistant to heat stress 4. Moreover, overexpression of Hsp70 in response to heat stress can protect against organ damage and lethality 4, 5, 6. Thus, induction of Hsp70 offers a potential target for the treatment of HS.

Heat shock protein 90 is a ubiquitous molecular chaperone that is involved in the folding, activation and assembly of many proteins, including key mediators of signal transduction and transcriptional regulation 7. Selective Hsp90 inhibitors, such as 17‐dimethylaminoethylamino‐17‐demethoxy‐geldanamycin (17‐DMAG), block the ATP‐binding site of Hsp90 and exert pleiotropic functions, including induction of the heat shock response 8. Previous studies have shown that these drugs can block the activity of certain pro‐inflammatory mediators in different cell types 9; for example, 17‐DMAG reduced the inflammatory response in atherosclerosis through up‐regulation of HSPs and the inhibition of pro‐inflammatory transcription factor activity 10. Therefore, pre‐treatment of rats with Hsp90 inhibitors may induce Hsp70 and improve cardiovascular dysfunction and survival during HS.

AMP‐activated protein kinase (AMPK) functions to monitor cellular energy status in response to nutritional environmental variations. Physiological or pathological stimuli that deplete cellular energy levels, such as prolonged exercise and oxidative stress, increase the AMP/ATP ratio, thereby activating AMPK 11. Once activated, AMPK leads to the conservation of intracellular ATP levels *via* multiple downstream pathways, including autophagy 12. Autophagy is a highly regulated process that involves the degradation of a cell's cytoplasmic macromolecules and organelles. In mammalian cells, this catabolic mechanism utilizes the lysosomal system and has a homeostatic function in normal cell growth and development, helping to maintain a balance among the synthesis, degradation and subsequent recycling of cellular products 13, 14. Although the exact role and relationship between autophagy and the heat stress response under stressful conditions remain to be determined, they cooperate in maintaining cellular homeostasis by facilitating appropriate folding of partially unfolded proteins or removing irreversibly damaged proteins to help the cell in coping with the cellular stress 15, 16, 17.

Here, we evaluated the effects of 17‐DMAG on Hsp70 and phosphorylated AMPK (pAMPK) in HS in rats.

## Materials and methods

### Experimental animals

Male Sprague–Dawley rats (300–350 g) were obtained from the National Laboratory Animal Breeding and Research Center of the National Science Council, Taiwan. Animal surgical procedures and handling were carried out as described previously 5. Handling of the animals was in accordance with the Guide for the Care and Use of Laboratory Animals published by the US National Institutes of Health (NIH Publication No. 85‐23, revised 1996). This study was approved by the National Defense Medical Center Institutional Animal Care and Use Committee, Taiwan.

### Experimental groups

**Figure 1:**
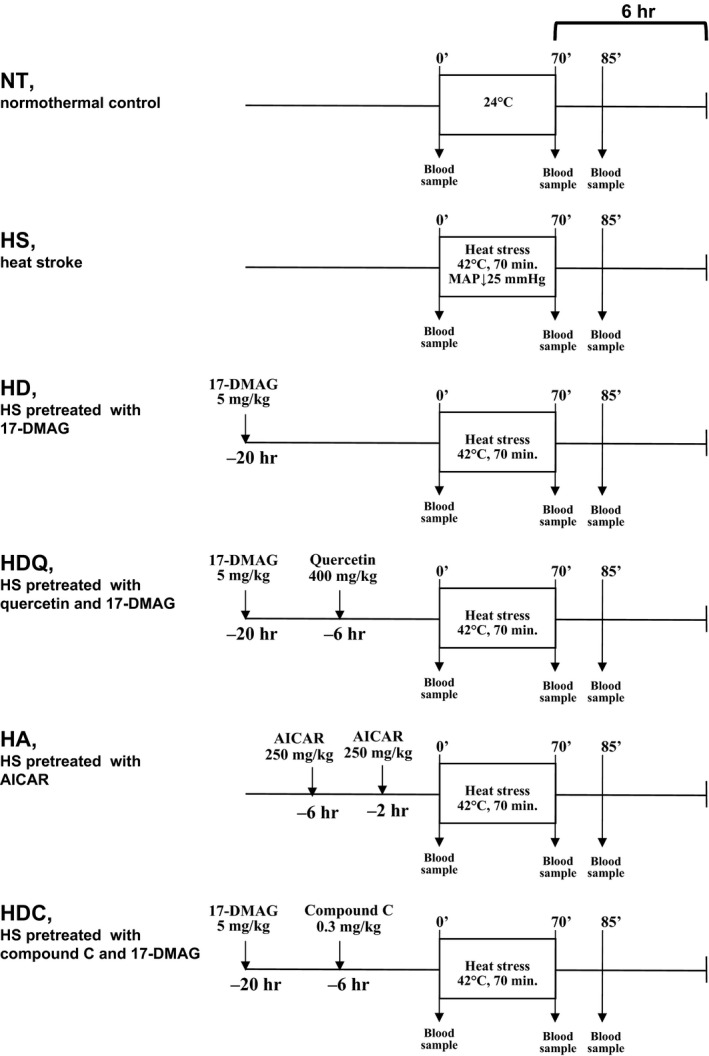
Experimental protocol. NT: normothermal control; HS: heat stroke; HD: HS pre‐treated with 17‐DMAG (5 mg/kg, i.p.); HDQ: HS pre‐treated with quercetin (400 mg/kg, i.p.) and 17‐DMAG (5 mg/kg, i.p.); HA: HS pre‐treated with AICAR (250 mg/kg); HDC: HS pre‐treated with compound C (0.3 mg/kg, i.p.) and 17‐DMAG (5 mg/kg, i.p.).

Rats under anaesthesia were randomized into six groups, as follows (Fig. 1): (*i*) the normothermic control (NT) group, wherein the core temperature was maintained at about 36°C with a heating chamber at a room temperature of 24 ± 1°C throughout the entire experiment; (*ii*) the saline‐treated HS group, in which HS was induced as described below; (*iii*) the 17‐DMAG pre‐treatment with HS (HD) group, in which rats received 17‐DMAG (5 mg/kg i.p.; InvivoGen, San Diego, CA, USA) for 20 hrs before heat stress; (*iv*) the 17‐DMAG and quercetin (Hsp70 inhibitor; Cayman Chemical, Ann Arbor, MI, USA) pre‐treatment with HS (HDQ) group, in which rats received quercetin (400 mg/kg i.p.) for 6 hrs and 17‐DMAG (5 mg/kg i.p.) for 20 hrs before heat stress; (*v*) the 5‐aminoimidizole‐4‐carboxamide riboside (AICAR; AMPK activator; Calbilchem, San Diego, CA, USA) pre‐treatment with HS (HA) group, in which rats received AICAR (250 mg/kg i.p.) for 2 and 6 hrs before heat stress and (*vi*) the 17‐DMAG and (6‐[4‐(2‐piperidin‐1‐yl‐ethoxy)‐phenyl])‐3‐pyridin‐4‐yl‐pyyrazolo[1,5‐a]‐pyrimidine (compound C; an AMPK inhibitor; Merck, Whitehouse Station, NJ, USA), pre‐treatment with HS (HDC) group, in which rats received compound C (0.3 mg/kg i.p.) for 6 hrs and 17‐DMAG (5 mg/kg i.p.) for 20 hrs before heat stress.

### Induction of heat stroke

Heat stroke was induced and animal handling was carried out as previously described 5, 18. Briefly, HS was induced by placing animals in a heating chamber (42°C) for approximately 70 min. The onset of HS was taken as the time at which the mean arterial pressure (MAP) fell to about 25 mmHg from the peak level and the rectal temperature (Tcore) was elevated to about 42°C. Survival of rats was monitored for 6 hrs and survival time was calculated at the interval between the onset of HS and animal death or killing at the experiment end with the Zoletil (15 mg/kg, i.p.; Virbac, Carros, France) overdose.

### Biochemical analysis

Whole blood (0.5 ml) was collected, and plasma levels of glutamic pyruvic transaminase (GPT) and creatinine were determined by spectrophotometry (Fiji DRI‐CHEM 303, Fuji photo Film Co., Tokyo, Japan).

### Determination of cytokines in plasma samples

Blood samples were collected immediately before the start of heat stress and at 70 and 85 min. after initiation of heat stress. The concentrations of tumour necrosis factor‐α (TNF‐α), interleukin (IL)‐6 and IL‐10 in plasma were determined with double‐antibody sandwich enzyme‐linked immunosorbent assays (ELISAs; R&D Systems, Minneapolis, MN, USA) according to the manufacturer's instructions.

### Histopathological analysis

Samples of the ileum were quickly excised, sliced into transverse or longitudinal sections and fixed in 10% neutral‐buffered formalin. The tissues were then embedded in paraffin blocks, and serial sections were stained with haematoxylin and eosin for microscopic evaluation (200× magnification). Morphological changes were assessed based on the pathologic assessment standards, including the extent and range of mucosal epithelial cell degeneration and necrosis, villus height and extent of structure damage.

### Western blotting

The liver tissue was ground in a mortar containing liquid nitrogen. The powdered tissue was then suspended in 1 ml of lysis buffer (50 mM 4‐(2‐Hydroxyethyl)piperazine‐1‐ethanesulfonic acid, N‐(2‐Hydroxyethyl)piperazine‐N'‐(2‐ethanesulfonic acid) (HEPES), 5 mM ethylenediaminetetraacetic acid, 50 mM NaCl, pH 7.5) containing protease inhibitors (10 μg/ml of aprotinin, 1 mM phenylmethylsulfonylfluoride and 10 μg/ml of leupeptin) and agitated at 4°C for 1 hr to evaluate protein expression. Nuclear and cytosolic extracts were prepared with a nuclear/cytosol fractionation kit (BioVision, Milpitas, CA, USA) according to the manufacturer's protocol.

Samples containing equal amounts of protein were loaded onto 10% SDS‐PAGE, subjected to electrophoresis and subsequently blotted onto nitrocellulose membranes (Millipore, Bedford, MA, USA). Membranes were blocked with Tris‐buffered saline (TBS), pH 7.4, containing 0.1% Tween 20 and 5% skim milk and then incubated overnight at 4°C with various primary antibodies in TBS containing 0.1% Tween 20. The antibodies included mouse anti‐Hsp70 antibody (1:1000 dilution; Stressgen Biotechnologies Co., Victoria, BC, Canada), anti‐heat shock factor‐1 (HSF‐1) antibody (1:1000 dilution; Santa Cruz Biotechnology, Santa Cruz, CA, USA), anti‐pAMPKα (Thr172) and anti‐AMPKα antibodies (Cell Signaling Technology, Danvers, MA, USA), anti‐Light chain 3 (LC3B) antibody (1:1000 dilation; Cell Signaling Technology) and mouse anti‐β‐actin antibody (1:2000 dilution; Sigma‐Aldrich, St. Louis, MO, USA). The membranes were incubated with horseradish peroxidase (HRP)‐conjugated secondary antibody (1:1000 dilution; Cell Signaling Technology), followed by enhanced chemiluminescence reagents (Pierce, Rockford, IL, USA). A bio‐imaging analyser (Fujifilm LAS‐4000; GE Healthcare Life Sciences, Mariborough, MA, USA) was used for visualization, and band densities were quantified by Image‐Pro software (Media Cybemetrics, Inc., Bethesda, MD, USA).

### Statistical analysis

Results are presented as the means ± S.E.M.s and were evaluated statistically by one‐way anova with Newman–Keuls multiple comparisons tests for the post‐hoc determination of significant differences. Differences with *P*‐values of less than 0.05 were considered significant.

## Results

### Effects of 17‐DMAG on tissue Hsp70 levels and HSF‐1 activation following HS

**Figure 2:**
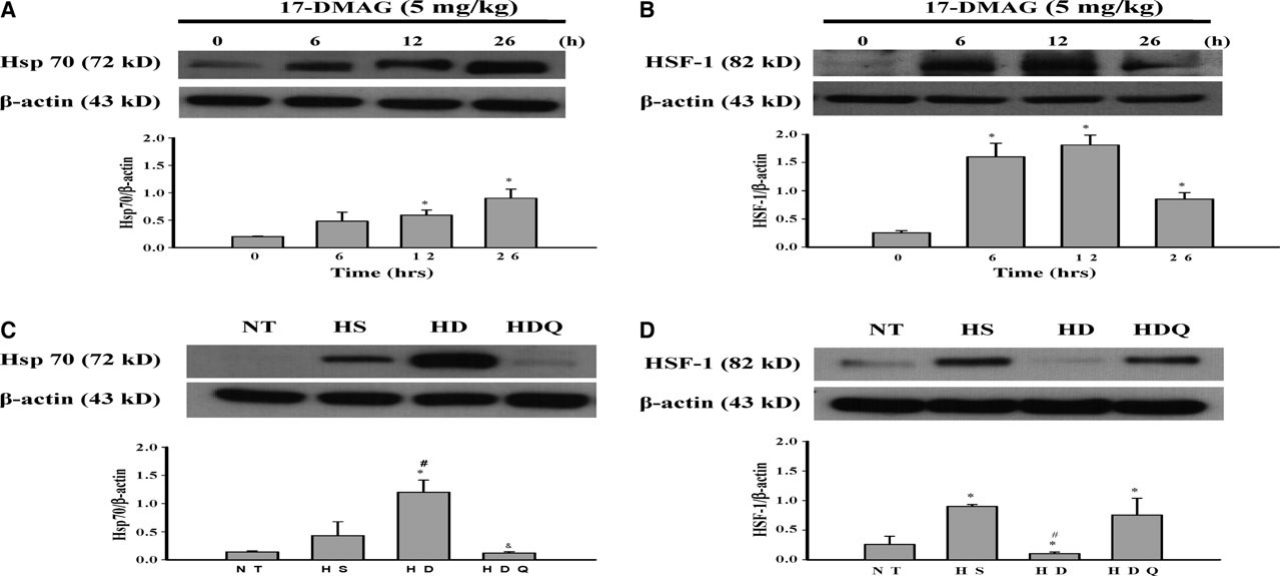
Effects of 17‐DMAG on Hsp70 and HSF‐1 expression following heat stroke. (**A** and **B**) Time‐dependent changes in Hsp70 and nuclear HSF‐1 expression in livers from rats after treatment with 17‐DMAG alone. Data are expressed as means ± S.E.M.s (*n* = 4). *P < 0.05 compared with 0 hr. (**C** and **D**) Effects of 17‐DMAG pre‐treatment on the expression of Hsp70 and HSF‐1 in the liver. Data are expressed as means ± S.E.M.s (*n* = 4), *P < 0.05 compared with the NT group, ^#^ *P* < 0.05 compared with the HS group, ^&^ *P* < 0.05 compared with the HD group.

In the liver, Hsp70 expression began to increase at 12 hrs and peaked at 26 hrs after treatment (Fig. 2A). Nuclear HSF‐1 expression began to increase at 6 hrs, peaked at 12 hrs and returned to baseline by 26 hrs after 17‐DMAG treatment (Fig. 2B). As shown in Figure 2C and D, compared with NT and HS rats, 17‐DMAG‐treated HS rats had significantly higher levels of Hsp70 in the liver. Moreover, nuclear HSF‐1 levels in the livers of HS group rats were significantly higher than those in the NT and HD groups; a similar pattern was observed for the HDQ group.

### 17‐DMAG attenuated HS‐induced physiological dysfunction

**Figure 3:**
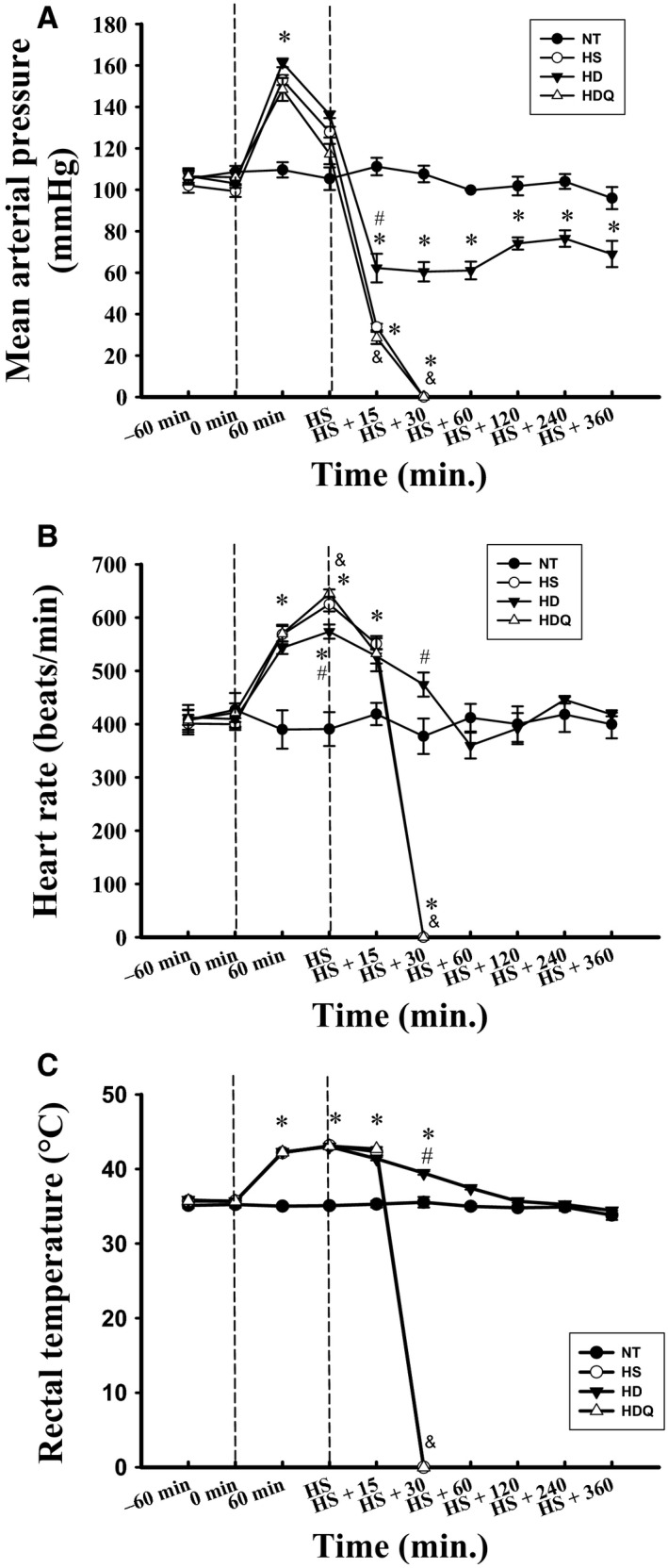
17‐DMAG attenuated heat stroke–induced physiological dysfunction. Effects of 17‐DMAG pre‐treatment on mean arterial pressure (MAP), heart rate and rectal temperature (Tcore). Data are expressed as means ± S.E.M.s (*n* = 6). *P < 0.05 compared with the NT group, ^#^ *P* < 0.05 compared with the HS group, ^&^ *P* < 0.05 compared with the HD group.

In the HS and HD groups, the MAP, heart rate and Tcore were all significantly higher at 60–70 min. after the start of heat stress than those in the NT group (Fig. 3). Compared with the NT group, the HS group showed significantly higher Tcore and heart rate values but a lower MAP at 85 min. after heat stress. Heat‐induced hypotension and tachycardia, but not hyperthermia, were significantly attenuated by 17‐DMAG pre‐treatment. The beneficial effects of 17‐DMAG were significantly suppressed by quercetin.

### 17‐DMAG attenuated HS‐induced inflammatory mediators

**Figure 4:**
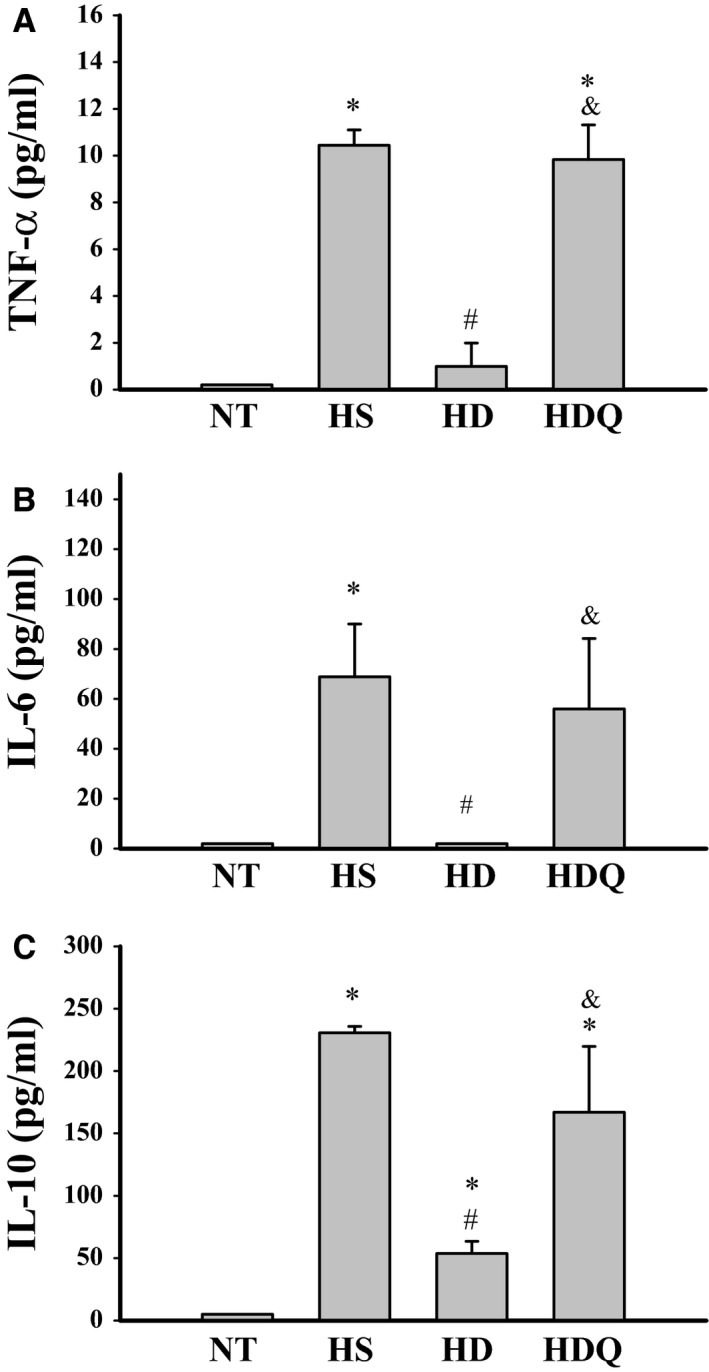
17‐DMAG attenuated heat stroke–induced inflammatory mediators. (**A**–**C**) Effects of 17‐DMAG pre‐treatment on plasma tumour necrosis factor (TNF)‐α, interleukin (IL)‐6 and IL‐10 levels. Data are expressed as means ± S.E.M.s (*n* = 5). *P < 0.05 compared with the NT group, ^#^ *P* < 0.05 compared with the HS group, ^&^ *P* < 0.05 compared with the HD group.

The basal plasma levels of TNF‐α, IL‐6 and IL‐10 were not significantly different among the four experimental groups. However, the plasma levels of these parameters in the HS group were significantly higher at 85 min. after the start of heat stress than those in the NT and HD groups (Fig. 4). Pre‐treatment with 17‐DMAG significantly attenuated the HS‐induced increase in plasma levels of these factors. In contrast, compared with the HD group, rats in the HDQ group had higher levels of pro‐inflammatory and anti‐inflammatory cytokines.

### 17‐DMAG attenuated HS‐induced intestinal injury

**Figure 5:**
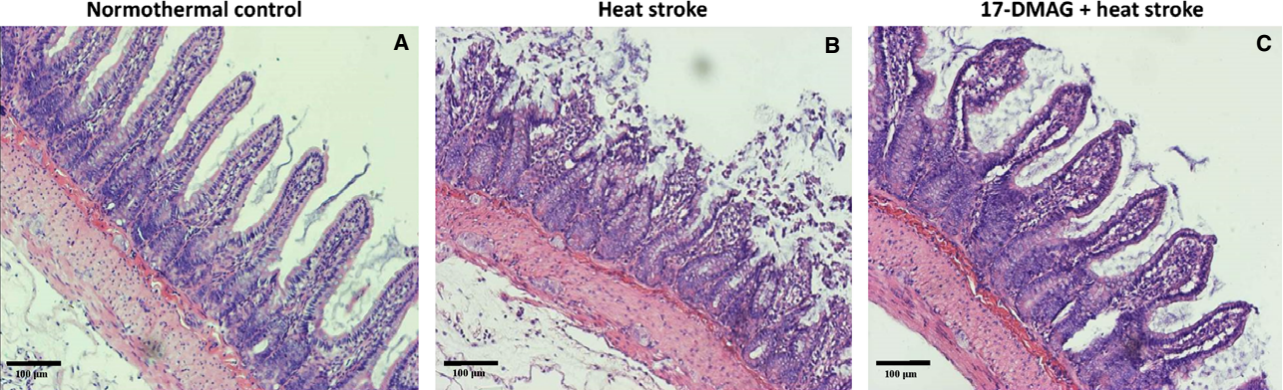
Pathological changes in the ileum of rats during heat stress. Photomicrographs of typical haematoxylin and eosin‐stained sections of the ileum. Animals were sacrificed at 85 min. after heat exposure for haematoxylin and eosin staining (magnification, 200×).

In the NT group, no marked damage was observed in the small intestinal mucosa (Fig. 5A). In the HS group, intestinal mucosa damage was characterized by marked mucosal disintegration of the lamina propria (Fig. 5B) compared with that in the NT group. This effect was attenuated by 17‐DMAG pre‐treatment, resulting in focal epithelial lifting from the lamina propria and vacuolation of the villi (Fig. 5C).

### 17‐DMAG attenuated increased plasma levels of organ injury indicators during HS

**Figure 6:**
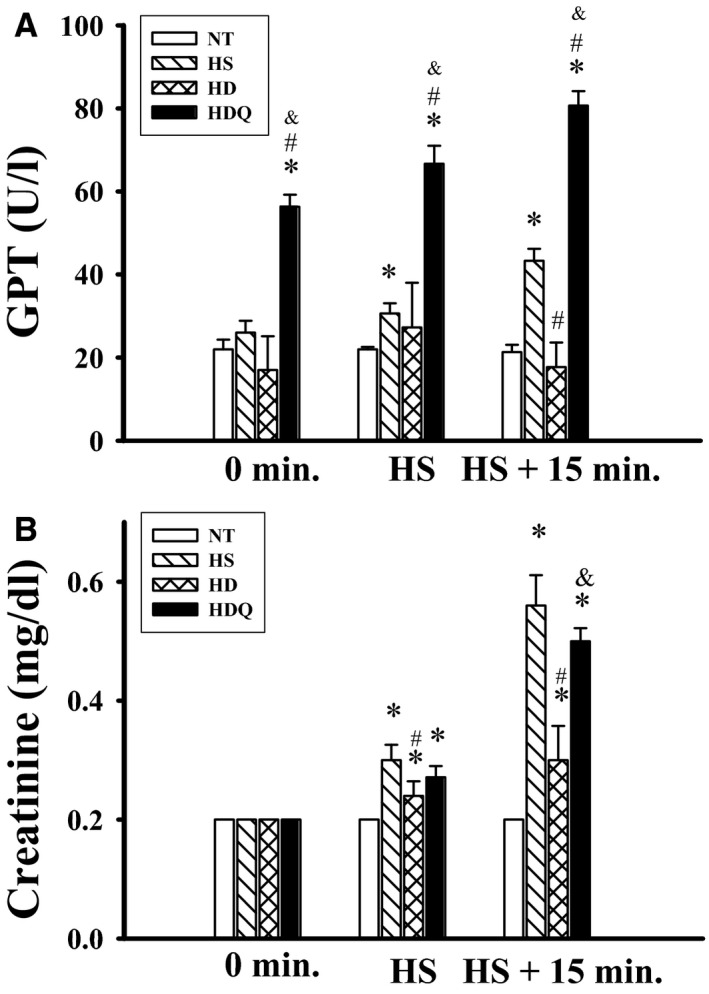
17‐DMAG attenuated increased plasma levels of organ injury indicators during heat stroke. Effects of 17‐DMAG pre‐treatment on plasma GPT (**A**) and creatinine (**B**). Data are expressed as means ± S.E.M.s (*n* = 6). *P < 0.05 compared with the NT group, ^#^ *P* < 0.05 compared with the HS group, ^&^ *P* < 0.05 compared with the HD group.

Compared with the NT group, the HS group had higher plasma levels of GPT and creatinine at 85 min. after heat exposure (Fig. 6). Heat‐induced increased plasma levels of GPT and creatinine were significantly reduced by pre‐treatment with 17‐DMAG, and these beneficial effects of 17‐DMAG were significantly attenuated by quercetin.

### 17‐DMAG pre‐conditioning increased pAMPK levels during HS

**Figure 7:**
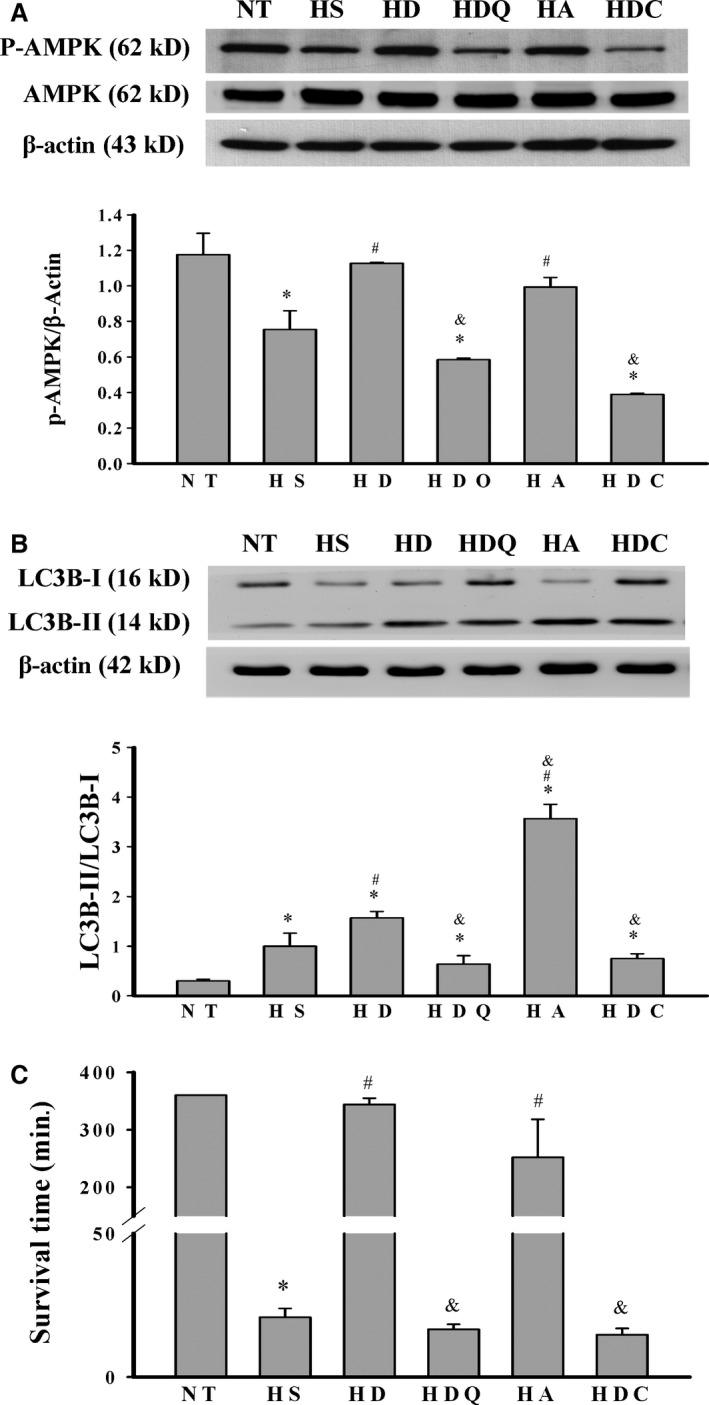
17‐DMAG pre‐conditioning increased pAMPK and autophagy‐related protein levels and survival times in heat stroke rats. Effects of 17‐DMAG pre‐treatment on pAMPK levels (**A**), LC3BII/LC3BI ratios (**B**) and survival times (**C**). Data are expressed as means ± S.E.M.s (*n* = 5). *P < 0.05 compared with the NT group, ^#^ *P* < 0.05 compared with the HS group, ^&^ *P* < 0.05 compared with the HD group.

Phosphorylated AMPK levels in the livers of rats were decreased after HS (Fig. 7). Compared with the HS group, the HD and HA group exhibited significantly higher hepatic pAMPK levels. These effects were significantly attenuated by pre‐treatment with quercetin or compound C.

### 17‐DMAG pre‐conditioning increased expression of the autophagy‐related protein LC3B

Compared with the NT and HS groups, the HD and HA groups exhibited higher LC3BII/LC3BI ratios in the livers of rats (Fig. 7B), and these increases were dramatically inhibited by pre‐treatment with quercetin or compound C.

### 17‐DMAG improved survival times after HS

The survival time of rats in the NT group was more than 360 min.; this was decreased to 21 ± 4 min. after the induction of HS in rats (Fig. 7C). However, the survival times of rats in the HD and HA groups were significantly increased to 344 ± 11 min. and 252 ± 66.2 min., respectively. In contrast, compared with rats in the HD group, rats in the HDQ and HDC groups showed reduced survival times.

### Involvement of AMPK in the protective effects of 17‐DMAG against HS

**Figure 8:**
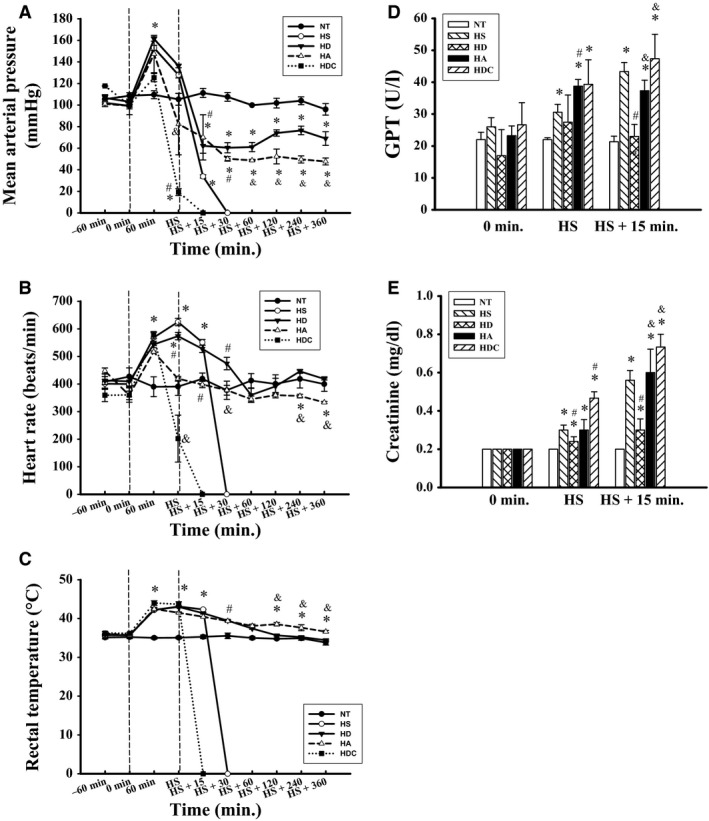
Involvement of AMPK in the protective effects of 17‐DMAG in heat stroke rats. Mean arterial pressure (MAP) (**A**), heart rate (**B**), rectal temperature (Tcore) (**C**), plasma GPT (**D**) and plasma creatinine (**E**) values were measured. Data are expressed as means ± S.E.M.s (*n* = 5). *P < 0.05 compared with the NT group, ^#^ *P* < 0.05 compared with the HS group, ^&^ *P* < 0.05 compared with the HD group.

As shown in Figure 8A–C, in the HDC group, MAP, heart rate and Tcore values were lower at 70–85 min. after the start of heat stress than those in the HS and HD groups. The MAP and Tcore values were similar between the HA and HD groups; however, the heart rate was lower in the HA group than in the HS and HD groups. Moreover, the HDC and HA groups had higher plasma levels of GPT and creatinine evaluated at 85 min. after heat exposure than those in the HD group (Fig. 8D and E).

## Discussion

In this study, we demonstrated, for the first time, that 17‐DMAG significantly attenuated hypotension, tachycardia, hepatic dysfunction and intestinal injury induced by HS in rats and prolonged survival times after HS. Our data also indicated that 17‐DMAG may function through induction of Hsp70 and pAMPK and that enhancing autophagy through up‐regulation of Hsp70 and pAMPK may be a potential therapeutic strategy for the treatment of HS.

Heat stroke can be effectively reproduced by exposing anesthetized rodents to a hot environment 19, 20; this causes splanchnic ischaemia and hypoxia, inducing generation of reactive oxygen species in the gut and increasing intestinal mucosal permeability to endotoxins. Endotoxemia can stimulate the production of pro‐inflammatory cytokines, which may trigger systemic inflammation and can cause inadequate coagulation, cellular ischaemia and damage and multi‐organ failure 19, 21. In contrast, in response to this pro‐inflammatory reaction, the body also produces an anti‐inflammatory response, including induction of IL‐10 22. In this study, we showed that pre‐treatment with 17‐DMAG significantly reduced heat‐induced inflammation and organ dysfunction and prolonged survival times. 17‐DMAG also attenuated IL‐10 levels. However, whether the inhibitory effects of 17‐DMAG are a result of a reduction in inflammatory cytokines remains to be determined.

Overexpression of Hsp70 in response to heat stress can protect against organ damage and lethality 4, 6, 23. Our current results demonstrated that pre‐treatment with 17‐DMAG before the start of heat stress significantly increased Hsp70 levels in the liver. Moreover, the protective effects of 17‐DMAG were attenuated when HS rats were pre‐treated with quercetin, an Hsp70 inhibitor, indicating that pre‐treatment with 17‐DMAG may improve survival by ameliorating organ injury during HS as a result of induction of Hsp70 overexpression. 17‐DMAG inhibits Hsp90 *via* binding to the N‐terminal domain of Hsp90 and disassociating the Hsp90‐HSF1 complex, resulting in activation of the transcription factor HSF‐1 24. Heat shock protein 70 is a transcriptional target of HSF‐1. Under physiological conditions, HSF‐1 remains a monomer in the cytosol. During heat stress, HSF‐1 is rapidly converted into its active form and initiates transcription and synthesis of Hsp70 after activation 25, 26, 27. In this study, the expression of nuclear HSF‐1 in livers of rats in the HD group was significantly lower than that in the HS and HDQ groups, whereas the expression of Hsp70 in the livers of rats in the HD group was significantly greater than that in the NT, HS and HDQ groups. One possible explanation of these conflicting results is that 17‐DMAG treatment may have activated HSF‐1 before the time‐points examined, resulting in elevation of Hsp70 before the pre‐hyperthermia time‐point. This is supported by our data showing that 17‐DMAG administration significantly enhanced the expression of nuclear HSF‐1 and Hsp70 in the livers of rats at 6–12 hrs and 12–26 hrs after treatment respectively. Further studies are needed to clarify the mechanisms through which 17‐DMAG regulates the expression of Hsp70 and HSF‐1.

The effects of heat stress on AMPK activation remain unclear 28. In this study, the phosphorylation of AMPK was reduced in the liver, suggesting that pAMPK inhibition was a universal response to HS. Hepatic pAMPK levels were significantly higher in the HD and HA groups than in the HS and HDQ groups, and administration of 17‐DMAG or the AMPK activator AICAR prevented HS‐induced death and heat stress‐induced pAMPK down‐regulation. Notably, the protective effects of 17‐DMAG were attenuated when HS rats were pre‐treated with the AMPK inhibitor compound C. Therefore, the positive regulation of pAMPK by Hsp70 may be a common mechanism involved in pAMPK induction after 17‐DMAG treatment.

In addition, AMPK activates intracellular ATP levels *via* autophagy 12. During autophagosome formation, endogenous LC3 is processed to LC3‐I, which is further converted into LC3‐II. The LC3II/LC3I ratio was markedly elevated following 17‐DMAG treatment in HS rats, and this phenomenon was reversed by compound C. Moreover, AICAR pre‐treatment mimicked the effects of 17‐DMAG on LC3II/LC3I in HS rats. These results suggested that 17‐DMAG effectively enhanced the HS‐induced autophagic protection effects in the liver through activation of AMPK. Our data revealed that overexpression of Hsp70‐enhanced HS‐induced autophagy after 17‐DMAG treatment; this phenomenon was reversed by quercetin. In addition, autophagy may contribute to the Hsp70‐mediated protective effects in HS rats, and 17‐DMAG prec‐onditioning may trigger autophagy *via* multiple signalling pathways involving Hsp70 and AMPK activation. We propose that 17‐DMAG may increase autophagic protective effects in the liver through Hsp70‐dependent AMPK activation, consistent with a previous report 29. However, other regulators may be involved in the protective effects of 17‐DMAG in heat stoke rats.

## Conclusion

Pre‐treatment with 17‐DMAG increased Hsp70 and pAMPK levels in the liver and blocked the HS response. 17‐DMAG also had anti‐inflammatory effects and induced autophagy in an AMPK‐ and/or Hsp70‐dependent manner in HS rats. These findings revealed the underlying mechanisms through which 17‐DMAG administration ameliorated the effects of HS, suggesting that 17‐DMAG may be a promising candidate drug for protection against HS.

## Conflicts of interest

The authors declare that they have no competing financial interests.

## Author contribution

Y.J.P., C.Y.Y. and Y.J.T. acquired data; Y.C.T., K.K.L., M.H.Y. and P.Y.C. contributed to the initial discussion of the project; Y.M.L. and P.Y.C. reviewed the article; Y.C.T. and P.Y.C. wrote the article.
